# Altered cerebral neurovascular coupling in medication-overuse headache: A study combining multi-modal resting-state fMRI with 3D PCASL

**DOI:** 10.3389/fnins.2023.1139086

**Published:** 2023-03-15

**Authors:** Xin Li, Mengqi Liu, Wenping Fan, Huan Xu, Zhiye Chen

**Affiliations:** ^1^Department of Radiology, Hainan Hospital of PLA General Hospital, Sanya, China; ^2^The Second School of Clinical Medicine, Southern Medical University, Guangzhou, China

**Keywords:** medication-overuse headache, resting-state fMRI, arterial spin labeling, neurovascular coupling, brain, magnetic resonance imaging

## Abstract

**Aim:**

Structural and functional changes in the brain have been identified in individuals with medication-overuse headache (MOH) using MRI. However, it has not been clearly established whether neurovascular dysfunction occurs in MOH, which could be elucidated by examining neurovascular coupling (NVC) from the viewpoints of neuronal activity and cerebral blood flow. The aim of this study was to investigate potential alterations in NVC function of the brain in individuals with MOH using resting-state functional MRI (rs-fMRI) and 3D pseudo-continuous arterial spin labeling (3D PCASL) imaging techniques.

**Methods:**

A total of 40 patients with MOH and 32 normal controls (NCs) were recruited, and rs-fMRI and 3D PCASL data were obtained using a 3.0 T MR scanner. Standard preprocessing of the rs-fMRI data was performed to generate images representing regional homogeneity (ReHo), fractional amplitude of low-frequency fluctuation (fALFF), and degree centrality (DC); cerebral blood flow (CBF) images were generated using 3D PCASL sequence data. These functional maps were all normalized into Montreal Neurological Institute (MNI) space, and NVC was subsequently determined on the basis of Pearson correlation coefficients between the rs-fMRI maps (ReHo, fALFF, and DC) and CBF maps. The statistical significance of differences between the MOH and NC groups in terms of NVC in different brain regions was established *via Z*-test. Further analysis was performed to examine correlations between NVC in the brain regions with NVC dysfunction and clinical variables among patients with MOH.

**Results:**

NVC mainly presented a negative correlation in patients with MOH and NCs. No significant difference between the two groups was detected in terms of average NVC over the entire gray matter area. However, several brain regions with significantly decreased NVC in patients with MOH compared to NCs were identified: the left orbital region of the superior frontal gyrus, the bilateral gyrus rectus, and the olfactory cortex (*P* < 0.05). A correlation analysis revealed that the DC of the brain regions with NVC dysfunction was significantly positively correlated with disease duration (*r* = 0.323, *P* = 0.042), and DC–CBF connectivity was negatively correlated with VAS score (*r* = −0.424, *P* = 0.035).

**Conclusion:**

The current study demonstrated that cerebral NVC dysfunction occurs in patients with MOH, and the NVC technique could function as a new imaging biomarker in headache research.

## Background

Medication-overuse headache (MOH), a consequence of regular overuse of acute or symptomatic headache medication, is defined as a type of secondary headache and most frequently occurs in the context of chronic migraine (CM; 2018; Wakerley, 2019). Although MOH is one of the top 20 causes of disability worldwide, with substantial socioeconomic consequences (Linde et al., 2012; Bendtsen et al., 2014; GBD 2015 Disease Injury Incidence Prevalence Collaborators, 2016), the neuromechanism of this disabling disease remains unknown. Therefore, revealing the possible pathophysiological mechanism might be helpful in developing better monitoring of and treatment for MOH.

Many advanced neuroimaging studies have demonstrated that pain not only causes abnormalities in brain structure but also induces alterations in cerebral function (Xie et al., 2016; Niddam et al., 2017; Shokouhi et al., 2018). These conventional neuroimaging techniques have identified altered gray matter volume, disrupted pain modulatory networks, and abnormal functional connectivity in MOH (Chanraud et al., 2014; Chen et al., 2016; Lai et al., 2016; Torta et al., 2016). However, a new neuroimaging technique that exploits neurovascular coupling (NVC; Fabjan et al., 2015) has also been applied to investigate abnormal brain metabolism in migraine. This technique is based on the coupling of microvascular blood flow and metabolic needs of the brain tissue, and may provide an innovative approach to detection of the pathophysiological mechanism of MOH.

NVC is responsible for ensuring an appropriate supply of blood to the brain and reflects the close temporal and regional linkage between neural activity and cerebral blood flow (CBF; Phillips et al., 2016). The NVC approach can involve assessment of both regional neural activity and regional CBF, thereby obtaining a more precise index for the measurement of microvascular blood flow and the metabolic needs of the surrounding brain tissue; this technique has been used in research on various disorders, such as Alzheimer's disease (AD), amyotrophic lateral sclerosis (ALS), chronic myofascial pain, and CM (Zlokovic, 2011; Osaki et al., 2018; Hu et al., 2019; Song et al., 2021). Therefore, assessment of NVC might be a complementary and comprehensive tool that can be used to observe dysfunction of the cerebral vasculature in MOH.

To date, NVC assessment has mainly employed one of the following techniques: (1) the combination of electroencephalography (EEG) with transcranial doppler (TCD) ultrasound, which had been applied in several previous studies (Rosengarten and Kaps, 2010; Rosengarten et al., 2012), but cannot assess NVC accurately at the same time because of the differences in machine patterns; (2) the combination of resting-state fMRI (rs-fMRI) and CBF analysis, which can provide a more feasible and accurate MRI measurement.

Advanced MRI technologies can help to provide better understanding of both perfusion and neuronal activity in the brain. 3D pseudo-continuous arterial spin labeling (3D PCASL) is an advanced perfusion imaging technique that can measure cerebral blood flow in the brain without an exogenous contrast agent, while rs-fMRI is based on spontaneous fluctuations in the blood oxygen level-dependent (BOLD) signal, which could be used to evaluate neuronal activity (Raimondo et al., 2021). BOLD-based analyses generate various parameters, such as regional homogeneity (ReHo), amplitude of low-frequency (0.01–0.08 Hz) fluctuation (ALFF), fractional ALFF (fALFF), and degree centrality (DC), which can be used to assess neuronal activity from different viewpoints. ReHo is designed to characterize the cohesiveness of resting activity in a given voxel with its neighboring voxels (Zang et al., 2004). The ALFF directly correlates with the intensity of spontaneous neural activity in the resting state, and fALFF further reduces the effects of physiological noise, which could represent the oxygen uptake ability of neurons and neuronal activity (Qiu et al., 2019). DC focuses on the functional relationships between a region and the rest of the brain over the entire connectivity matrix of the brain at the voxel level (Gao et al., 2016). Although all signals in rs-fMRI are derived from blood flow, the related fMRI parameters (ReHo, fALFF, and DC) reflect spontaneous fluctuations in the BOLD signal from a microscopic viewpoint, while CBF reflects true cerebral blood flow from the macroscopic viewpoint. Consequently, we can use the 3D PCASL and rs-fMRI techniques to construct an NVC model in order to assess the correlation between cerebral perfusion and neuronal activity in MOH.

To better explore the underlying brain alterations occurring in MOH, in the current study, we aimed to test the hypothesis that abnormal NVC in the brain occurs in patients with MOH. To address the hypothesis, NVC was determined from the Pearson correlation coefficients between the rs-fMRI map (ReHo, fALFF, and DC) and the CBF map over the whole brain in patients with MOH and normal controls (NCs). Additionally, a correlation analysis was performed to examine the relationship between NVC and clinical variables for the brain regions with NVC dysfunction in patients with MOH.

## Methods

### Subjects

This study was approved by the ethics committee of the Chinese PLA General Hospital, and written informed consent was obtained from all participants.

In the current study, 40 patients with MOH were recruited from the headache clinic of the Chinese PLA General Hospital. A total of 32 NCs, matched on age and sex, were recruited from among hospital staff members and their relatives. The inclusion criterion for patients with MOH was that they should meet the diagnostic criteria in the International Classification of Headache Disorders, third edition (beta version; ICHD-3 beta; 2018); the inclusion criterion for NCs was that they should never have had any primary headache disorder or other types of headache in the past year. All subjects also needed to meet the following conditions: (1) be right-handed; (2) avoid alcohol, nicotine, caffeine, and other substances for at least 12 h before MRI examinations; (3) be normotensive ( ≤ 140/90 mmHg); and (4) for patients with MOH, be in the interictal stage at least 3 days after a migraine attack. The exclusion criteria for all participants were as follows: brain tumor, definite white matter lesion, cerebrovascular disease such as infarction or malacia, psychiatric disorders, and regular use of a psychoactive or hormone medication. No subjects enrolled in the study had any cardiovascular or metabolic disorders.

All subjects were evaluated on several neuropsychological scales, including the Hamilton Anxiety Scale (HAMA), the Hamilton Depression Scale (HAMD), and the Mini-Mental State Examination (MMSE) for evaluation of cognitive function. Disease duration (DD), Visual Analog Scale (VAS) scores, and Migraine Disability Assessment (MIDAS) questionnaire scores were also used to measure pain intensity and to evaluate level of disability for patients with MOH only.

### MRI acquisition

All MR data were obtained using a GE 3.0 T MR system (DISCOVERY MR750, GE Healthcare, Milwaukee, WI, USA). A conventional eight-channel quadrature head coil was used, and foam padding was used to limit head movement. MR sequences were carried out as follows: (1) conventional axial T2WI and diffusion-weighted imaging to exclude subjects with cerebral lesions, such as infarction, malacia, and brain tumor; (2) a three-dimensional T1-weighted fast spoiled gradient recalled echo (3D T1-FSPGR) sequence with voxel size 1^*^1^*^1 mm over the whole brain; (3) resting-state fMRI, collected using a gradient echo-planar imaging (EPI) sequence (TR/ TE = 2,000/30 ms, flip angle = 90°, slice thickness = 4 mm, slice gap = 1 mm, FOV = 24 × 24 cm, Matrix = 64 × 64); 180 continuous EPI functional volumes were acquired axially over 6 min, during which subjects were instructed to relax, keep their eyes closed, stay awake, remain still, and clear their heads of all thoughts; (4) cerebral perfusion imaging, obtained using 3D pseudo-continuous arterial spinal labeling (3D PCASL) with the following settings: TR/TE = 5,128/15.9 ms, FOV = 20 × 20 cm, x, y matrix = 1,024 × 8 (spiral acquisition), slice thickness = 3.0 mm, labeling duration = 1.5 s, and post-labeling delay (PLD) time = 1.5 s. The 50 slices for each CBF image were automatically generated using Functional Tools (version 9.4.05) after PCASL scanning (Chen et al., 2018).

### Data processing

Resting-state functional images and CBF images were processed using Statistical Parametric Mapping 12 (SPM12; http://www.fil.ion.ucl.ac.uk/spm) and DPABI (a toolbox for data processing and analysis of brain imaging; V5.1; Chao-Gan and Yu-Feng, 2010), which were run under MATLAB 7.6 (MathWorks, Natick, MA, USA).

The fMRI data preprocessing steps were as follows (Chen et al., 2016; Hu et al., 2019): (1) discarding of the first 10 volumes of each functional time course; (2) slice timing; (3) head motion correction; (4) spatial normalization; (5) calculation of ReHo, DC, and fAlff maps; and (6) Z-score transformation and spatial smoothing.

Cerebral blood flow (CBF) images were obtained using an ASL sequence and processed as follows (Shang et al., 2020): the raw CBF images were coregistered with the raw 3D T1WI (3D T1-FSPGR) and were then normalized to Montreal Neurological Institute (MNI) space using the forward deformation field generated from the segments of 3D T1 images; the normalized CBF images were then Z-scored by subtracting the global mean and dividing by the standard deviation (SD).

**Figure 1:**
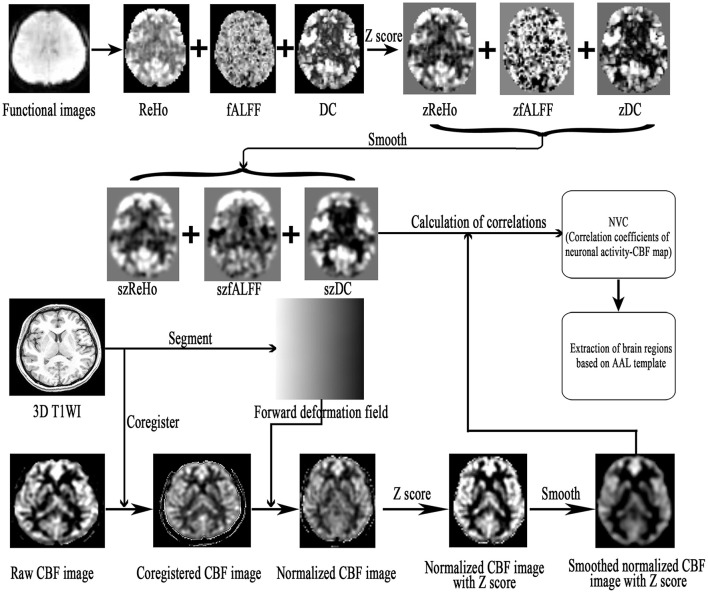
. A flowchart outlining the analysis of neurovascular coupling (NVC) in the brain.

The NVC preprocessing steps were as follows (Figure 1). (1) The resting-state fMRI data were processed as described in previous studies, and the zReHo, zfALFF, and zDC (z, z-score) subsequently generated. (2) The CBF images were processed to generate the zCBF. (3) zReHo, zfALFF, zDC, and zCBF were smoothed using a 6 × 6 × 6 mm Gaussian kernel full width at half maximum (FWHM). (4) The NVC maps (ReHo-CBF, fALFF-CBF, and DC-CBF) were calculated by using the Pearson method to compute correlations between maps of neuronal activity (ReHo, fALFF, and DC) and perfusion (CBF) images for the MOH and NC groups. (5) Voxel-wise comparison of NVC was performed using the SPM 12 software, with age and gender as covariates. The minimal number of contiguous voxels was based on the expected cluster size. Significance was set at a *P*-value of < 0.05 with false discovery rate (FDR) correction. (6) The NVC index (consisting of correlation coefficients between neuronal activity and perfusion maps; Hu et al., 2019) was extracted from the NVC maps using the Automated Anatomical Labeling (AAL) atlas (Rolls et al., 2015).

### Statistical analysis

Quantitative data following a normal distribution are presented in the form mean ± SD, and non-normally distributed quantitative data are presented in the form of median (minimum, maximum). Comparisons of quantitative variables were performed with independent-samples *t*-tests, and comparisons of qualitative variables were performed with the chi-square test. The correlation analysis was performed with the Pearson method for normally distributed data and with the Spearman method for non-normally distributed data. Comparisons of correlation coefficients were performed using the *Z*-test. These analyses were carried out using MedCalc (V19.0.4), and a *P*-value of < 0.05 was considered to indicate a statistically significant difference.

## Results

### Demographic and clinical characteristics

Demographic and clinical characteristics of the participants are presented in Table 1. There was no statistically significant difference in Aage, gender, or MMSE scores between the two groups (*P* > 0.05). Patients with MOH had significantly higher HAMA (18.73 ± 8.82) and HAMD (19.78 ± 11.94) scores compared with NCs (HAMA score: 10.19 ± 2.98; HAMD score 8.61 ± 4.33; *P* < 0.05).

**Table 1:** Demographic and clinical characteristics of the subjects.

	**MOH**	**NC**	***T*-value**	***P*-value**
Num (F/M)	40 (32/8)	32 (20/12)	2.68 (χ^2^)	0.10
Age	41.95 ± 9.82	41.34 ± 10.89	0.25	0.80
HAMA	18.73 ± 8.82	10.19 ± 2.98	5.24	< 0.0001
HAMD	19.78 ± 11.94	8.61 ± 4.33	5.48	< 0.0001
MMSE	25.35 ± 6.08	25.53 ± 6.24	0.13	0.90
DD (years)	20.00 (3.00, 50.00)	NA		
VAS	8.00 (5.00, 10.00)	NA		
MIDAS	135.00 (0, 260.00)	NA		

MOH, medication-overuse headache; NC, normal control; HAMA, Hamilton Anxiety Scale; HAMD, Hamilton Depression Scale; MMSE, Mini-mental State Examination; DD, disease duration; VAS, Visual Analog Scale; MIDAS, Migraine Disability Assessment questionnaire; NA, not available.

### Altered ReHo–CBF over all cerebral gray matter regions in MOH

**Figure 2:**
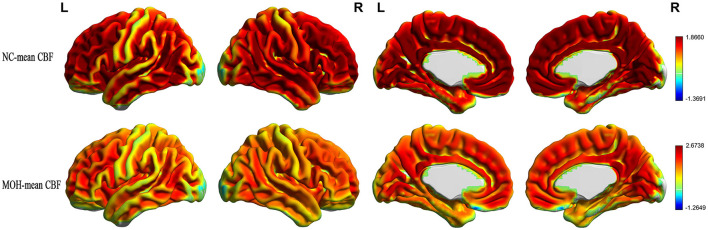
Mean CBF maps for the NC and MOH groups, showing no obvious difference in distribution over the entire gray matter level.

**Figure 3:**
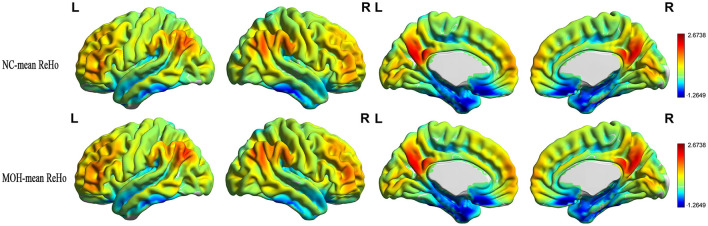
Mean ReHo maps for the NC and MOH groups, showing no obvious difference in distribution over the entire gray matter level.

**Figure 4:**
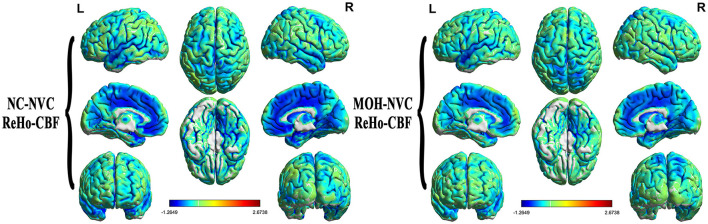
Neurovascular coupling (NVC) in normal controls (NCs) and patients with medication-overuse headache (MOH), mainly indicating negative correlation between regional homogeneity (ReHo) and cerebral blood flow (CBF) over the entire gray matter area.

**Figure 5:**
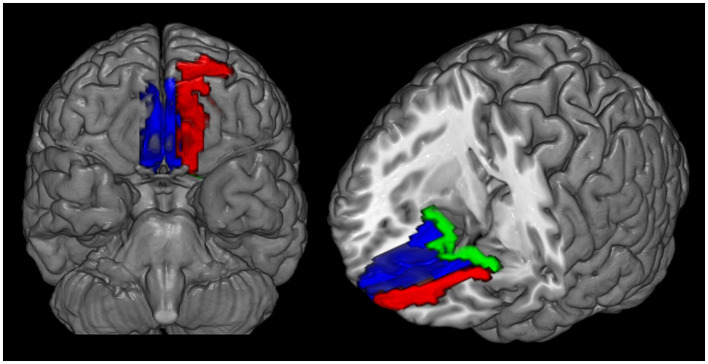
Decreased ReHo–CBF connectivity located in the left orbital region of the superior frontal gyrus (red region), the bilateral gyrus rectus (blue region), and the olfactory cortex (green region) in patients with medication-overuse headache (MOH) compared with normal controls (NCs).

Mean CBF and mean ReHo maps for NCs and patients with MOH are shown in Figures 2, 3, respectively. Figure 4 illustrates the fact that NVC, as indexed by ReHo–CBF, mainly showed a negative correlation over all whole gray matter regions in NCs and patients with MOH. Moreover, there was no significant difference in ReHo–CBF over the entire gray matter level between the MOH (*r* = 0.458) and NC (*r* = 0.422) groups (*Z*-value = 0.177, *P* = 0.860). However, further regional NVC analysis determined that brain regions with significantly decreased ReHo–CBF connectivity in the MOH group compared with the NC group were located in the left orbital region of the superior frontal gyrus, the bilateral olfactory cortex, and the bilateral gyrus rectus (Figure 5); the corresponding correlation coefficients can be seen in Table 2.

**Table 2:** Brain regions with altered NVC over the whole gray matter in MOH.

**NVC**	**Anatomic region**	***r***		***Z*-value**	***P*-value**
		**MOH**	**NC**		
ReHo-CBF	ORBsup.L	−0.298	−0.685	−2.141	0.032
	Olfactory_L	−0.638	−0.903	−2.950	0.003
	Olfactory_R	−0.640	−0.891	−2.702	0.007
	Rectus_L	−0.294	−0.738	−2.295	0.010
	Rectus_R	−0.334	−0.707	−2.155	0.031
fALFF-CBF	ORBsup.L	−0.366	−0.719	−2.102	0.036
	Olfactory_L	−0.630	−0.906	−3.085	0.002
	Olfactory_R	−0.630	−0.881	−2.577	0.010
	Rectus_L	−0.250	−0.726	−2.684	0.007
	Rectus_R	−0.329	−0.706	−2.164	0.030
DC-CBF	ORBsup.L	−0.353	−0.725	−2.214	0.027
	Olfactory_L	−0.506	−0.878	−3.270	0.001
	Olfactory_R	−0.532	−0.872	−3.027	0.003
	Rectus_L	−0.209	−0.725	−0.285	0.004
	Rectus_R	−0.276	−0.704	−2.389	0.017

ReHo, regional homogeneity; fALFF, fractional amplitude of low-frequency fluctuation; DC, degree centrality; CBF, cerebral blood flow; MOH, medication-overuse headache; NC, normal control; NVC, neurovascular coupling; ORBsup.L, left orbit part of superior frontal gyrus; Olfactory, olfactory cortex; Rectus, gyrus rectus; L, left; R, right.

### Altered fALFF–CBF over all cerebral gray matter regions in MOH

**Figure 6:**
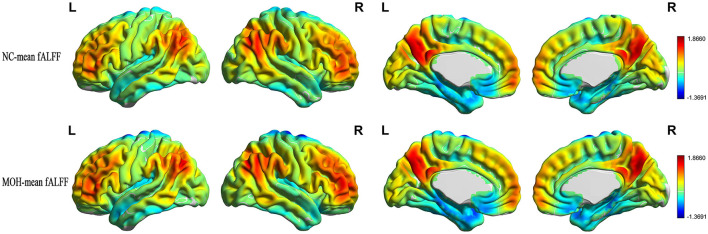
Mean fALFF in normal controls (NCs) and patients with medication-overuse headache (MOH).

**Figure 7:**
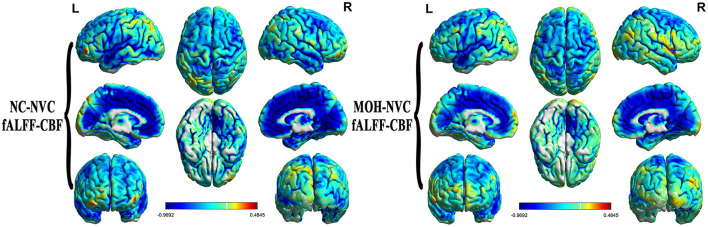
fALFF–CBF connectivity, mainly indicating a negative correlation in normal controls (NCs) and patients with medication-overuse headache (MOH), and also showing no significant difference over the entire gray matter level.

Mean fALFF in the NC and MOH groups can be seen in Figure 6. The fALFF–CBF measure mainly presented a negative correlation in both NCs and patients with MOH (Figure 7). There was no significant difference in fALFF–CBF connectivity over the entire gray matter level between the MOH (*r* = 0.475) and NC (*r* = 0.504) groups (*Z*-value = 0.154, *P* = 0.878). However, there was significantly decreased fALFF–CBF connectivity in the MOH group, compared with fALFF–CBF of NCs, in the left orbital region of the superior frontal gyrus, the bilateral olfactory cortex, and the bilateral gyrus rectus (Figure 5; *P* < 0.05).

### Altered DC–CBF over all cerebral gray matter regions in MOH

**Figure 8:**
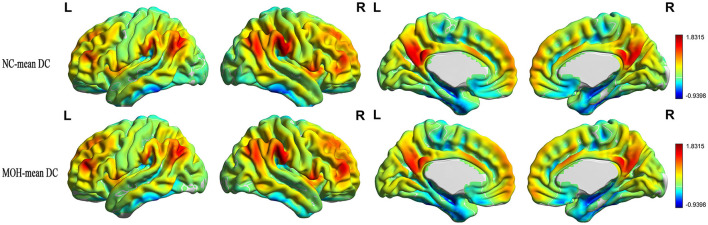
Mean DC in normal controls (NCs) and patients with medication-overuse headache (MOH) over the entire gray matter level.

**Figure 9:**
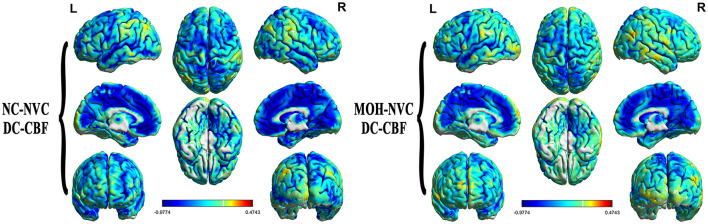
DC–CBF connectivity of NCs and patients with MOH, mainly indicating a negative correlation over the entire gray matter level.

Figure 8 presents the mean DC among NCs and patients with MOH over all gray matter regions. DC–CBF connectivity of NCs and patients with MOH mainly presented a negative correlation over all gray matter (Figure 9). However, mean DC–CBF over all gray matter regions showed no significant difference between the NC (*r* = 0.494) and MOH (*r* = 0.492) groups (*Z*-value = 0.011, *P*-value = 0.992). Further comparisons of specific brain regions identified significantly decreased DC–CBF connectivity in the MOH group compared to the NC group in the left orbital region of the superior frontal gyrus, the bilateral olfactory cortex, and the bilateral gyrus rectus (Figure 5; *P* < 0.05).

### Correlations of resting-state fMRI variables and NVC in brain regions with altered NVC with clinical characteristics in MOH

**Figure 10:**
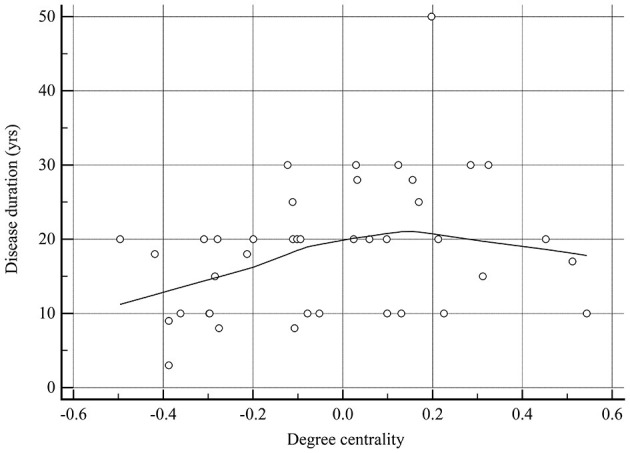
There was a significant positive correlation between degree centrality of the brain regions with altered NVC and disease duration in patients with MOH.

As shown in Table 3, the only significant positive correlation with disease duration was that of DC of brain regions with altered NVC (*r* = 0.323, *P* = 0.042; Figure 10). ReHo and fALFF of brain regions with altered NVC did not have any positive significant correlations with any clinical variables in the MOH group (*P* > 0.05).

**Table 3:** Correlation between the resting-state fMRI variables extracted from the brain regions with altered NVC and the clinical characteristics in MOH.

	**ReHo**		**fALFF**		**DC**		**CBF**	
	***r***	***P***	***r***	***P***	***r***	***P***	***r***	***P***
DD	0.121	0.459	0.125	0.443	0.323	0.042	−0.317	0.063
VAS	−0.100	0.557	−0.036	0.827	0.247	0.125	−0.154	0.377
HAMA	0.068	0.678	−0.011	0.947	−0.075	0.646	−0.133	0.447
HAMD	−0.104	0.525	−0.083	0.609	−0.156	0.335	−0.074	0.672
MIDAS	−0.078	0.633	−0.207	0.199	−0.251	0.118	−0.056	0.750
MMSE	0.018	0.911	−0.158	0.331	−0.052	0.750	0.093	0.593

DD, disease duration; VAS, Visual Analog Scale; HAMA, Hamilton Anxiety Scale; HAMD, Hamilton Depression Scale; MIDAS, Migraine Disability Assessment questionnaire; MMSE, Mini-mental State Examination; ReHo, reagional homogeneity; fALFF, fractional amplitude of low-frequency fluctuation; DC, degree centrality; CBF, cerebral blood flow.

**Figure 11:**
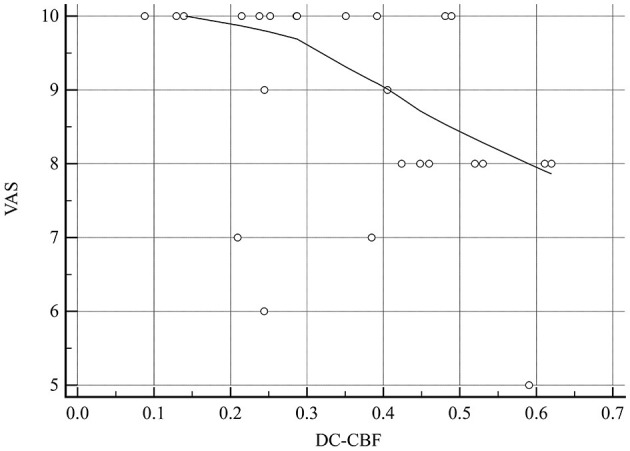
There was a significant negative correlation between DC–CBF of the brain regions with altered NVC and VAS score in patients with MOH.

The DC–CBF value of brain regions with altered NVC presented a significant negative correlation with VAS score (*r* = −0.424, *P* = 0.035; Table 4 and Figure 11). ReHo–CBF and fALFF–CBF of brain regions with altered NVC showed no significant negative correlations with any clinical variables in the MOH group (*P* > 0.05).

**Table 4:** Correlation between NVC connectivity of the brain regions with altered NVC and the clinical characteristics in MOH.

	**ReHo-CBF**		**fALFF-CBF**		**DC-CBF**	
	***r***	***P***	***r***	***P***	***r***	***P***
DD	0.023	0.934	0.327	0.137	−0.286	0.166
VAS	−0.054	0.842	0.190	0.397	−0.424	0.035
HAMA	−0.052	0.849	0.236	0.291	−0.291	0.158
HAMD	−0.291	0.274	−0.018	0.936	−0.363	0.074
MIDAS	0.252	0.347	0.311	0.159	−0.237	0.254
MMSE	0.369	0.160	0.081	0.721	0.130	0.534

DD, disease duration; VAS, Visual Analog Scale; HAMA, Hamilton Anxiety Scale; HAMD, Hamilton Depression Scale; MIDAS, Migraine Disability Assessment questionnaire; MMSE, Mini-mental State Examination; ReHo, regional homogeneity; fALFF, fractional amplitude of low-frequency fluctuation; DC, degree centrality; CBF, cerebral blood flow.

## Discussion

In the current study, we assessed NVC *via* a combination of rs-fMRI brain maps and CBF maps as measures of neuronal activity and perfusion, respectively. In this article, we have reported a series of NVC findings whose interpretation may help suggest some new hypotheses on the potential neurological pathology of MOH.

This study revealed that neuronal activity (ReHo, fALFF, and DC) was mainly negatively correlated with brain perfusion (CBF) on the voxel level in patients with MOH. Under physiological conditions, perfusion is tightly coupled with brain metabolism during rest, and functional brain hubs exhibit high CBF, suggesting that highly connected functional hubs are more metabolically active and exhibit higher levels of energy consumption (Liang et al., 2013; Galiano et al., 2020). However, in the present study, neuronal activity mainly showed a negative correlation with brain perfusion in patients with MOH, indicating that the normal physiological condition was disturbed, and the balance between neuronal activity and metabolic supply was probably disrupted from the NVC viewpoint.

In addition, we further explored multiple NVC indices (ReHo–CBF, fALFF–CBF, and DC–CBF), which showed no significant difference between patients with MOH and NCs over the entire cerebral gray matter level. However, regional NVC analysis demonstrated that values of ReHo–CBF, fALFF–CBF, and DC–CBF were lower in the MOH group than that in the NC group in the following brain regions: the left orbital region of the superior frontal gyrus (ORBsup.L), the bilateral olfactory cortex, and the bilateral gyrus rectus. Therefore, regional NVC analysis might be much more sensitive for the evaluation of neuronal vascular activity than overall cerebral NVC assessment in MOH. Meanwhile, changes in NVC could be interpreted in terms of its structural basis, that is, neurovascular units (NVUs), which are mainly composed of neurons, vascular cells, and glial cells (Phillips et al., 2016). Under this view, decreased NVC in patients with MOH implies possible damage to or degeneration of NVUs, which may be a consequence of repeated migraine attacks. Therefore, further NVC evaluation might provide more valuable information to develop an understanding of the neural mechanism of MOH.

In this study, decreased NVC in ORBsup.L was confirmed in patients with MOH; this might be associated with abnormal metabolism in MOH. The majority of rs-fMRI studies have revealed that patients with migraine show functional alterations in the superior frontal gyrus compared with NCs (Tian et al., 2021; Cao et al., 2022; Yang et al., 2022). Furthermore, one 18-FDG-PET study has initially reported hypometabolism in the orbitofrontal cortex, the bilateral thalamus, and other regions. Interestingly, after medication withdrawal, other regional metabolic changes were found to normalize in this study, with the exception of continued hypometabolism in the orbitofrontal region (Fumal et al., 2006), which might be related to the fact that the orbitofrontal cortex plays a role in addictive disorders. Moreover, the results of structural imaging studies have revealed abnormalities, including lower orbitofrontal cortex volume and thickness (Chong, 2019). Therefore, the decreased NVC in ORBsup.L observed in the current study is consistent with the abovementioned previous functional and structural studies, which could be used to explain MOH pathogenesis.

An abnormal condition of the olfactory cortex may be associated with the development of osmophobia, leading to fear, aversion, or psychological hypersensitivity to odors in patients with migraine (Harriott and Schwedt, 2014; Rocha-Filho et al., 2015). Chen et al. reported increased ReHo values in the olfactory cortex of patients with CM, using in measure in which attention was paid only to neural activity, with perfusion changes being ignored (Chen et al., 2019). However, the current study found decreased NVC in the olfactory cortex in patients with MOH; the inconsistency in these results may be due to the consideration of perfusion status and different stages of migraine. In contrast, a “neurolimbic” model has also been proposed, which represents dynamic bidirectional influence of pain and mood (such as anxiety and depression), and the activation of the limbic system, making patients with migraine susceptible to stress and emotional reactions (Maizels et al., 2012). It is well-known that the olfactory cortex is subordinate to the limbic system; this may explain why patients with MOH had significantly higher HAMA and HAMD scores compared with NCs. In addition, dysfunction of the limbic system also plays a role in chronic transformation of migraine (Chen et al., 2019); therefore, the pattern of decreased NVC indicates that the limbic system may play a role in the evolution from CM to MOH. Based on all these factors, the presence of abnormal NVC may bear further investigation in terms of understanding the mechanism of MOH.

The gyrus rectus is situated medially to the olfactory sulcus at the ventromedial edge of the frontal lobe, and is specifically connected to auditory cortex neurons in the convexity of the superior temporal gyrus according to animal research (Müller-Preuss et al., 1980). A PET study demonstrated that the gyrus rectus may be a component of the circuit that mediates certain specific cognitive and emotional functions (Andreasen et al., 1995). The decreased level of NVC in the gyrus rectus implies an abnormal association with auditory cortex neurons, further contributing to a disturbance in the auditory sensory modalities of patients with MOH (Harriott and Schwedt, 2014). A significant decrease in ALFF–CBF has been found in the left gyrus rectus in patients with CM (Hu et al., 2019); however, decreased NVC (including ReHo–CBF, fALFF–CBF, and DC–CBF) of the bilateral gyrus rectus was confirmed in this study. This result indicates, using the NVC approach, that the gyrus rectus may be a conjunctive connection of the brain regions involved in CM and MOH development, but the exact neuromechanism should be investigated more precisely in future.

Finally, correlation analysis revealed that DC in the regions of the brain that were positive for NVC dysfunction was significantly positively correlated with disease duration, and DC–CBF connectivity showed a negative correlation with VAS score. Previous research has demonstrated that nociceptive stimuli may result in NVC dysfunction (Wolf et al., 2011; Uchida et al., 2017); the current study further supports the possibility that the abnormal NVC observed in MOH may be a consequence of the strength and extent of previous migraine attacks. However, in the current study, we did not find significant correlations between ReHo–CBF, or fALFF–CBF values and clinical variables. This is again unsurprising, since DC indexes the number of direct connections for a given voxel in a network, reflecting its functional connectivity within the brain network without requiring *a priori* selection (Gao et al., 2016). Therefore, non-invasive NVC techniques could be considered as an objective strategy for the evaluation of pain status in MOH.

Our study has several limitations. First, this study employed a cross-sectional observational design; a longitudinal study should be performed to observe the evolution of MOH from CM. Second, it would be better to estimate altered NVC in patients with MOH before and after medication withdrawal. Third, this study only examined NVC changes in the interictal stage; future studies should assess different time points (i.e., the interictal and ictal stages) in the migraine cycle to provide more information about patients' NVC status. Fourth, the signal-to-noise ratio (SNR) of rs-fMRI and 3D PCASL is relatively low; therefore, it will be important to improve the resolution of these techniques in future work.

## Conclusion

The current study demonstrated that cerebral NVC dysfunction occurs in patients with MOH, and can be detected by multi-modal rs-fMRI combined with a 3D PCASL technique; NVC could function as a new imaging biomarker in future research on headache.

## Data availability statement

The raw data supporting the conclusions of this article will be made available by the authors, without undue reservation.

## Ethics statement

Institutional Review Board of the Chinese PLA General Hospital approved the research protocol and the procedures conformed to the tenets of the Declaration of Helsinki. Written informed consent for participation was not required for this study in accordance with the national legislation and the institutional requirements.

## Author contributions

ZC: conception, design, and revision of the manuscript for intellectual content. ML: acquisition of data. XL: analysis of data. XL, WF, and HX: drafting of the manuscript. All authors read and approved the final manuscript.
