# Corrigendum: Altered cerebral neurovascular coupling in medication-overuse headache: a study combining multi-modal resting-state fMRI with 3D PCASL

**DOI:** 10.3389/fnins.2023.1195725

**Published:** 2023-04-13

**Authors:** Xin Li, Mengqi Liu, Wenping Fan, Huan Xu, Zhiye Chen

**Affiliations:** ^1^Department of Radiology, Hainan Hospital of PLA General Hospital, Sanya, China; ^2^The Second School of Clinical Medicine, Southern Medical University, Guangzhou, China

**Keywords:** medication-overuse headache, resting-state fMRI, arterial spin labeling, neurovascular coupling, brain, magnetic resonance imaging

**Incorrect Affiliation**

In the published article, there was an error in affiliation(s) 1. Instead of “The Second School of Clinical Medicine, Southern Medical University, Guangzhou, China”, it should be “Department of Radiology, Hainan Hospital of PLA General Hospital, Sanya, China”.

In the published article, there was an error in affiliation(s) 2. Instead of “Department of Radiology, Hainan Hospital of Chinese PLA General Hospital, Sanya, China”, it should be “The Second School of Clinical Medicine, Southern Medical University, Guangzhou, China”.

The authors apologize for this error and state that this does not change the scientific conclusions of the article in any way. The original article has been updated.

**Additional Affiliation(s)**

In the published article, there was an error regarding the affiliation 1 for Xin Li and Zhiye Chen. As well as having affiliation(s) 1, they should also have *The Second School of Clinical Medicine, Southern Medical University, Guangzhou, China*.

The authors apologize for this error and state that this does not change the scientific conclusions of the article in any way. The original article has been updated.

